# The Effects of the Global Economic Recession and a Reduced Alcohol Tax on Hospitalizations Due to Alcohol-Attributed Diseases in Taiwan

**DOI:** 10.3390/ijerph14060580

**Published:** 2017-05-30

**Authors:** Chen-Mao Liao, Chih-Ming Lin

**Affiliations:** 1 Department of Applied Statistics and Information Science, Ming Chuan University, Taipei 333, Taiwan; cmliao@mail.mcu.edu.tw; 2 Department of Healthcare Information and Management, Ming Chuan University, Taipei 333, Taiwan

**Keywords:** alcohol-attributed disease, morbidity, economic recession, time-series, ARIMA

## Abstract

This study is to assess the effects of the 2008 economic crisis and a 2009 alcohol tax reduction on alcohol-related morbidity for men of different socioeconomic statuses in Taiwan. Admissions data for the period from 2007 to 2012 for men aged 24–59 years in 2007 was retrieved from the National Health Insurance Research Database. With stratification over three income levels, an interrupted time-series analysis examining the effects of the crisis and taxation reduction on incidence rates of hospitalization for alcohol-attributed diseases (AADs) was employed. The low income group showed a significant (*p* < 0.05) change in the rate of AAD-related hospitalizations in July 2008; specifically, an abrupt 7.11% increase that was then sustained for several months thereafter. In contrast, while the middle income group exhibited a significant 22.9% decline in the rate of AAD-related hospitalizations over the course of the crisis, that downward trend was gradual. The reduction of the alcohol tax resulted in increased rates of AADs among both the low and high income groups. The economic recession and the reduction of the alcohol tax resulted in an increased rate of AAD among low income men.

## 1. Introduction

Beginning in the third quarter of 2008, a major global economic crisis struck many developed industrial countries. By April 2009, the International Monetary Fund estimated that banks and other financial institutions around the world had lost four trillion US dollars [1]. The World Bank also estimated a consequent rise in global unemployment of around 30 million, resulting in additional deaths during the recession period [2]. Judging from earlier experiences of financial crises in various parts of the world, stresses associated with rising unemployment, poverty, and social insecurity will lead to upward trends in suicide rates, as well as to increases in the prevalence of various psychiatric illnesses [3,4,5,6,7,8]. Previous studies have found that alcohol consumption, meanwhile, rises along with income, indicating that alcohol is a normal good [9,10,11]. Bor et al. interviewed adults in the US between 2006 and 2010 to assess changes in their alcohol use and found that the prevalence of alcohol consumption significantly declined during the Great Recession [12]. On the other hand, during an economic downturn, people may feel stress and decreased levels of happiness even without losing their jobs, and such feelings could lead to increased levels of alcohol consumption as well as to changes in the types of alcohol consumed [13,14]. Stuckler et al. reported that increases of more than 3% in unemployment resulted in increases of at least 28.0% in death rates due to alcohol abuse for those younger than 65 years of age during the downturn in various European countries. The aforementioned findings raise the question of whether alcohol is an inferior good or a normal good according to economic conditions [15].

In Asia, an economic resurgence was halted by the outbreak of the global financial crisis, which was itself triggered by the collapse of Lehman Brothers in the third quarter of 2008. Due to the economy’s high dependence on foreign trade, the sharp contraction in demand in Western markets caused Taiwan’s exports to decline steeply, which in turn caused industrial production to plummet and resulted in negative growth for the economy as a whole. Compared with neighboring export-oriented Asian countries, Taiwan was not only the first to suffer a plunge in exports, but also suffered a greater decline than any of the others. According to official government statistics, the rate at which the gross domestic product (GDP) of Taiwan was increasing dropped gradually from 6% in 2007 to 1.9% in 2009 [16]. The GDP fell by 4% in the second quarter of 2009, leading to a clear spike in the unemployment rate (from 3.9% in 2007 to 5.9% in 2009) [17].

Domestically produced rice spirits have always been the most popular alcoholic beverage in Taiwan. In June 2009, a new official policy enforced industries whereby the tax rate on rice alcohol was reduced. This constituted a 74% reduction of the sale price, which subsequently led to the volume of rice spirits sold per year to increase from 10 million bottles to 85 million bottles. Previous studies have established the negative effects of beverage alcohol taxes and prices on sales and drinking outcomes [18,19,20,21,22]. Nonetheless, the magnitude of the effect posed by alcohol taxation is largely dependent upon the taxation amount, income levels, and drinking culture [23,24].

Alcohol consumption and the resulting harm may vary according to demographics. One national survey previously reported that the alcohol use prevalence rates during a recent one-month period for men and women aged 12–64 years in 2005 were 41.2% and 14.5%, respectively [25]. Socioeconomic differences in severe alcohol-related harm have also been reported to be substantial, with these differences widening still further over the past two decades [26]. Previous studies reported that the income-mortality disparity was wider for alcohol-related mortality than for all-cause mortality [27,28]. A systematic review suggested that lower socioeconomic status leads to higher mortality for alcohol-attributable causes compared with all causes [29]. Relatedly, although the effects of the financial crisis on alcohol-related problems have been explored on several occasions, most of the previous studies have focused on alcohol-related behaviors or mortality and have considered the entire sample as the unit of analysis without taking into account how differences in socioeconomic status might affect the frequency and characteristics of health problems [12,13,14,15,30]. Additionally, individual and collective responses to the crisis that could have led to different health outcomes may also have been influenced by specific cultural contexts [31,32].

A systematic review reported that several related psychological theories (i.e., the stress-response-dampening theory, self-medication theory, income-effect theory, and non-working time theory) could explain how these crisis-triggered consequences could increase the levels of alcohol consumption and alcohol-related health outcomes. The study also suggested that in many countries (i.e., Eastern Europe, Sweden, Finland, Russian, and the USA), the psychological distress mechanism was observed mainly in men. The tighter budget constraint mechanism seemed to play a role in all population subgroups across all countries [33]. In addition to its export-oriented economy, Taiwan has specific drinking customs such as the consumption of rice spirits rather than simply wine or distilled spirits as Western countries would. Our study attempts to provide more evidence in different cultural contexts. Moreover, in light of the differential effects on various socio-economic groups, further inquiry addressing the differential effect on health outcomes will help identify vulnerable targets, thus allowing for the development of effective prevention strategies [34]. Taiwan is heavily dependent on the global economy, and is therefore vulnerable to economic recessions and trade tariffs. However, policy makers have paid little attention to how recessions affect the health of either the broad population or specific socioeconomic subgroups in Taiwan. Therefore, this study aims to evaluate the impact of the 2008 financial crisis and the 2009 tax reduction on alcohol-attributed disease (AAD) on different socioeconomic subgroups in Taiwan.

## 2. Materials and Methods

### 2.1. Data Measurement

Taiwan launched a single-payer National Health Insurance Program (NHIP) in March of 1995. Approximately 99% of Taiwan’s 23 million people have subsequently been enrolled in this program. The National Health Insurance Administration (NHIA) created the National Health Insurance Research (NHIR) database to collect data from the NHIP to facilitate research. The admissions data analyzed in this study was retrieved from the National Health Insurance Research Database (NHIRD) claims files. Specifically, data for 2007–2012 from three NHIP research databases, namely, the “registry for beneficiaries” (RB) database, the “inpatient expenditure by admissions” (IEA) database, and the “monthly claims summary for inpatient claims” database, was analyzed. The following information was obtained from the relevant health certificates included in the three claims datasets: personal identification number (PIN), gender, cause of disease, date of birth, date of admission, and insurance premium. In Taiwan, the labor force and the consumption of alcohol are dominated by working-age men aged 24–64 years. We focused on the economic impact on men. By selecting all the registered men with birth years from 1948 to 1983 (i.e., those aged 24–59 years old in 2007), a total of 6,859,847 men were included as the subjects of the study.

The primary diagnoses for each admission are coded in the NHIR database according to the International Classification of Diseases, Ninth Revision, using the International Classification of Diseases, 9th Revision, Clinical Modification codes. Our definitions for AADs (diseases for which excessive drinking is 100% responsible) were based on a comprehensive review of the literature [35,36,37,38]. We counted admissions which suffered from AADs including alcoholic liver disease, alcohol psychosis, alcohol abuse, alcohol dependence syndrome, alcoholic polyneuropathy, alcoholic cardiomyopathy, alcoholic gastritis, acute alcohol poisoning, and excessive blood level of alcohol. Because of the high accessibility of hospitals here, it was possible to include almost all the patients in Taiwan with severe alcoholic disorders in our study.

Data from the IEA database for 2007–2012 showed a total of 285,185 episodes of hospitalization were due to AADs in the cohort. Using individual PINs, we also linked the study cohort to the 2007 RB database to retrieve personal information regarding insurance premiums. For each participant, we used the paid insurance premium level in 2007 as a surrogate for personal income (incomes fall into one of the brackets of the premium schedules, and are the basis on which the paid premium is calculated), which in turn allowed us to categorize all the participants into low, middle, and high income groups, i.e., those with monthly incomes lower than 17,280 New Taiwan Dollars (the first quartile), those with monthly incomes of 17,280–33,300 (the second and third quartile), and those with monthly incomes of >33,300 (the fourth quartile). The number of admissions was counted for every month according to the admission date of hospitalization. The sex-age-income-specific populations were used as a denominator to calculate the monthly incidence rates of hospitalization according to the RB database information. As an outcome variable, each monthly incidence rate of hospitalization suffered due to AADs was divided further by an adjusted factor to avoid confounders resulting from changes in health policies or fluctuations in hospital service volumes. The adjusted factors were calculated in terms of the monthly incidence rates of hospitalization for all causes divided by the initial monthly incidence rates in 2007. The method for determining the adjusted incidence rate of hospitalization (AIRH) was used in a recent study [37]. The figure of monthly AIRH of AADs over the 6-year period and a one-step-ahead forecast which uses current and previous observations to predict the next value according to minimum mean square error was created.

### 2.2. Statistics Analysis

The study period yielded a series of 72 monthly observations of AIRH. A total of 18 of those observations included pre-interventional data (as the economic crisis in Taiwan did not occur until July 2008 with a manifestation of a sharp decline in GDP), while 12 observations were included in the first post-interventional period. The second post-interventional period was composed of 42 monthly observations from July 2009 to December 2012, following the reduction of the alcohol tax rate (i.e., the 30th month of data observation). Accordingly, two effects of the intervention were explored with regard to the occurrence of AADs in the study. Given a large number of repeated observations, we used an interventional analysis with noise series that followed a seasonal autoregressive integrated moving average (ARIMA) model, which is ideally suited for assessing the impact of an intervention on a stationary or non-stationary time series [39,40]. Firstly, by defining the 19th month of data observation as the time point at which the intervention began, a model for the time-series was specified that assessed monthly variations among the 18 pre-interventional observations. The autocorrelation function (ACF) and the partial autocorrelation function (PACF) were used to identify possible long-term trends and other regularities in the series. Furthermore, the unit-root test was performed with an augmented Dickey-Fuller test to identify stationarity for this series. Secondly, a temporary or permanent interventional component was added when an adequate model for the stochastic behavior of the series was identified, resulting in a full impact assessment model. The interventional components were also examined during the month of the intervention, as well as from the first to tenth month after the intervention. The potential lack-of-fit tests of these models were determined using Box–Ljung statistics. Finally, the statistics appropriate models were selected with Akaike’s information criterion based upon the maximum likelihood method. Please refer to the Technical Appendix for details of the model specification, model diagnosis, and parameter estimation. SAS version 9.1 (SAS Institute Inc., Cary, NC, USA) was used for storage and aggregation of the data, and all SRIMA analyses were performed with R version 3.2.5 (R Foundation for Statistical Computing, Vienna, Austria, 2016).

## 3. Results

**Figure 1:**
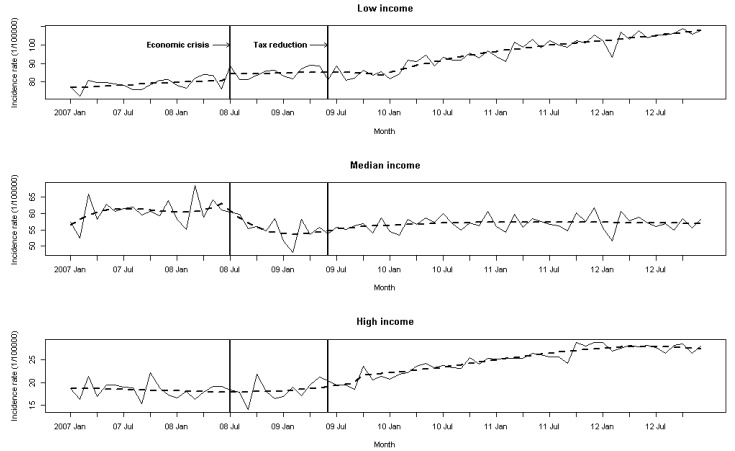
Monthly alcohol-attributed incident rates of hospitalization (per 100,000 persons) and a one-step ahead forecast with economic and tax intervention.

Our analysis found obvious differences in the AIRH for AADs among the three income groups. The annual averages for the low, middle, and high income groups in 2007 were 78.3 per 100,000 persons, 60.4 per 100,000, and 18.6 per 100,000, respectively. Unlike the low and high income groups, the middle income group showed a continuous reduction in the rate during the 2008–2009 period. The combined rate for the low and middle income groups was approximately two times higher than the combined rate for the middle and high income groups during the study period (Table 1). The monthly AIRH for AADs over the 6-year period and a one-step ahead forecast are shown in Figure 1. Different AIRH trends can be seen among the income groups. Visually, we found an obvious upward trend post-July 2008 for the low and high income groups. The monthly AIRH showed relative stability in the middle-income group, though an abrupt decline was seen after July 2008. Monthly variations were apparent in the series for all three groups, with prominent declines in January or February (when the Chinese lunar New Year holiday occurs), and these variations should be taken into account in modeling the series.

The appropriate statistical intervention or non-intervention models with income-specific monthly AIRH for AADs as dependent variables were identified as follows:

### 3.1. Model Identification

#### 3.1.1. Low Income

(1) $$y_{t}=\omega_{1}\;S_{19}+\frac{\omega_{2}B^{6}}{1-\delta_{2}B}S_{30}+\frac{1-\theta_{12}B^{12}}{1-B^{12}}a_{t}$$

$$S_{19}=\{\begin{array}{c} 0,\;\;if\;t<19\\ \;1,\;\;if\;t\geq 19\; \end{array}$$

$$S_{30}=\{\begin{array}{c} 0,\;\;if\;t<30\\ \;1,\;\;if\;t\geq 30\; \end{array}$$

where $y_{t}$ is the AIRH in observation *t*; $\omega_{1},\;\;\omega_{2}$, and $\delta_{2}$ are the intervention effective coefficient parameters; and the abrupt permanent effect is a step function beginning in the 19th and the 36th observation (that is, there was no lag for the permanent effect after the first intervention and a 6 month delay before the gradual permanent effect after the second intervention). *B* is the backshift operator, $a_{t}$ is white noise, and $\theta_{12}$ is the first-order annual moving average parameter. The estimates (SE) of $\omega_{1},\;\;\omega_{2},$ $\delta_{2}$, and $\theta_{12}$ are 5.072 (1.349), 0.977 (0.229), 0.970 (0.017), and 0.647 (0.138). $\omega_{1}$ and $\omega_{2}$ are 5.072 (1.349) and 0.977 (0.229) and are the first and second intervention effective coefficient parameters, respectively, for the AADs. The noise follows a $SARIMA(\left(0,0,0\right),{\left(0,1,1\right)}_{12})$ model. The residuals from the augmented model did not differ from white noise (Box-Ljung Q-statistics (with 18 months) = 22.82 (*p* = 0.155)). This specification was accepted as a model for monthly measurement. The parameters were all statistically significant (*p* < 0.05). The residual ACFs did not exhibit any model inadequacy. The above results imply that the first and second interventions induced a significant rise in AIRH in July 2008 (i.e., when the economic crisis began) and December 2009 (that is, there was a six-month lag after the tax intervention), respectively.

#### 3.1.2. Middle Income

(2) $$y_{t}=\;\frac{\omega_{1}}{1-\delta_{1}B}I_{\left(19,28\right)}+\frac{\left(1-\theta_{1}B\right)\left(1-\theta_{12}B^{12}\right)}{\left(1-B\right)\left(1-B^{12}\right)}a_{t}$$

$$I_{\left[19,\;28\right]}=\{\begin{array}{c} 1,\;\;\;if\;19\leq t\leq 28\\ 0,\;\;\;o.w \end{array}$$

The noise follows a $SARIMA(\left(0,1,1\right),{\left(0,1,1\right)}_{12})$ model, which is adjusted with a monthly and annual difference. The estimates (SE) of $\omega_{1}$, $\delta_{1}$, $\theta_{1}$, and $\theta_{12}$ are −1.042 (0.329), 0.924 (0.051), 0.805 (0.094), and 0.606 (0.134), respectively. The parameters are all statistically insignificant (*p* < 0.05). Those results imply that only the first intervention induced a significant gradual permanent decline in AIRH from July 2008 to April 2009 (i.e., the 28th observation). The residuals from the augmented model did not differ from white noise (Box-Ljung Q-statistics (with 18 months) = 15.54 (*p* = 0.485)).

#### 3.1.3. High Income

(3) $$y_{t}=\frac{\omega_{2}B^{3}}{1-\delta_{2}B}S_{30}+\frac{\left(1-\theta_{1}B\right)\left(1-\theta_{12}B^{12}\right)}{\left(1-B\right)\left(1-B^{12}\right)}a_{t}$$

The estimates (SE) of $\omega_{2}$, $\delta_{2}$, $\theta_{1}$, and $\theta_{12}$ are 0.561 (0.267), 0.939 (0.047), 0.797 (0.089), and 0.621 (0.125), respectively. The selected intervention model presents gradual permanent changes in the level of the outcome series, which is adjusted with a monthly and annual difference. The noise follows a $SARIMA(\left(0,1,1\right),{\left(0,1,1\right)}_{12})$ model. The effect is a step function beginning in the 33rd observation.

The above results imply that only the second intervention induced a significant gradual increase in AIRH in September 2009 (that is, there was a three-month lag after the tax intervention). The residuals from the augmented model did not differ from white noise (Box-Ljung Q-statistics (with 18 months) = 12.01 (*p* = 0.743)).

### 3.2. ARIMA Analyses

The Low/Middle income groups showed significant (*p* < 0.05) changes in the AIRH for AADs after the economic intervention. Results from the interrupted time-series analysis are presented in Table 2. In the low income group, the permanent model indicated an upward trend after the two interventions, which differed from the trend in the pre-intervention period that was noted in subsequent years. The AIRH for AADs showed a 7.11% increase in July 2008 compared to the 12-month pre-intervention period and a 39.74% increase by December 2009. On the other hand, the analysis for the middle income group indicated a significant 22.9% decline in the AIRH that began in July 2008 and was subsequently sustained for the next ten months. The tax reduction significantly raised the AIRH for AADs for the high income group, with a permanent increase of 47.69% in the rate occurring in September 2009.

## 4. Discussion

The study found that the 2008 global financial crisis was associated with a rise in alcohol disorders in Taiwan. The economic recession may result in adverse health effects due to alcohol consumption within a specific population. The finding is consistent with previous studies that were conducted in different cultural contexts. A Spanish study documented substantial increases in alcohol-related disorders among primary care attendees during an economic recession [41]. Harhay et al. reported that during a crisis, economic stressors had a stronger impact on alcohol-related outcomes than before the crisis among white British adults [42]. Mulia et al. reported that middle-aged Americans had a greater risk of drunkenness and alcohol-related problems during the 2008 to 2009 recession [43]. Economic hardship, unemployment, job insecurity, and the lack of a regular living wage all have important effects on health and the demand for health care [44,45]. Unemployment is associated with an increased risk of alcohol-related hospitalization or mortality. A recent study showed that job loss among middle-aged individuals during the economic recession was positively associated with becoming a hazardous drinker in European countries [46]. The postulated mechanisms through which job loss may affect health include stress associated with financial strain and the loss of psychosocial assets [47,48]. During an economic crisis, the number of households in high debt and the rates at which evictions and repossessions of homes occur both increase. Nonetheless, the impacts of economic crises and unemployment on alcohol consumption and alcohol-related health problems were different on subgroups [49,50]. Kalousova and Burgard suggested heterogeneity in the pathways that connect hardship experiences and different health behaviors [51]. Our study also could not support the psychological distress mechanism across all population subgroups in the Asian context.

We found evidence that the impact of an economic recession on alcohol disorders may have income disparity, most notably among socioeconomically disadvantaged men. Tarkiainen et al. considered that the income disparity linked to mortality statistics originated from alcohol-related deaths among the working aged population [52]. A Korean study reported that an increased alcohol-related mortality gap between social groups was attributable to increased alcohol consumption by socially disadvantaged middle-aged men after the economic crisis [53]. In contrast, Bor et al. found that white Americans with higher household incomes were more likely than those with lower incomes to engage in frequent binge drinking during the financial crisis [12]. It is a reasonable expectation that low income will correlate with a low level of education. According to annual human resources reports in Taiwan, from 2007 to 2009 the unemployment rates for men with secondary school level education and below increased from 3.7% to 7.1%, while the unemployment rates for men with college level education and above increased from 3.8% to 5.8% [53]. As domestically produced rice spirits are inexpensive and easy to obtain, these alcoholic beverages are always popular among the low socioeconomic population in Taiwan. Given our findings, these figures appear to corroborate our inference that people with social disadvantages may experience more severe unemployment risks and drinking disorders simultaneously, during the recession. In the eyes of the government, the impact of a financial crisis on health can be worse when social protection retrenches, following fiscal austerity ignited by an economic crisis [6]. The health and welfare system should target resources and care management to avoid or mitigate social and cost-related crises. Evidence also suggests that undesirable health effects can be ameliorated with strong social safety nets [34]. The government should foresee a grave picture for future economic events.

On the other hand, this study speculates that alcohol may be a normal good for middle-class men, which implies that their alcohol consumption, and corresponding alcohol-related conditions, will fall in response to declining incomes associated with the recession. Compared to the low income group, we think that more expensive alcohol beverages (e.g. wine or distilled spirits) may be consumed more frequently in higher income groups. We believe that these assumptions regarding alcohol as a normal/inferior good may not necessarily be generalized across different socioeconomic populations as this phenomenon may be related to the way in which different income classes view alcohol purchases (i.e., their acceptance of high-priced and low-priced alcohol). Additionally, the possibility that disadvantaged men would purchase spurious or illegally produced alcohol on the black market during times of hardship cannot be ruled out. This potential trend could in turn increase the possibility of alcohol-related health events. No significant increased admissions among the higher income groups during the economic recession were observed, although it should be noted that the admissions in high income men increased by 9% from 2008 to 2009. We also suspect that good health and social support among more socioeconomically advantaged members of the population may attenuate their risk of severe adverse outcomes, even if their alcohol use increases during or after an economic crisis.

A recent cross-sectional study reported that alcohol tax policy may have influences on the time trend of the hospitalization rate due to alcohol [37]. Our study verifies this negative relationship between taxes and alcohol-related morbidity. In addition, the study found that the impact of taxation is light for the middle class. This null result implies the fluctuation of alcohol prices may have less influence on the drinking behavior of middle-income men than on other men. We also found that the influences of the change in taxation on the hospitalization rates for drinking disorders were not evident until three or six months after the policy change itself was implemented in the second quarter of 2009. This delayed effect implies that, at least in the Asian context, the adverse outcomes are chronic and may be caused by increased binge drinking due to the reduction in the overall price of alcohol. Nonetheless, whether or the impacts of two interventions can be independent is controversial. The economic recession may have delayed these effects for years. According to annual human resources reports from Taiwan [54], the increase in the unemployment rate of men began in August 2008 and ceased in early 2010 during the period of time when our study was conducted. It is possible that the increase in admissions after 2009 could be attributed to the recession, in addition to the effects of the tax decrease. If that is true, the adverse effects caused by the recession could be delayed and the impacts of tax may be overestimated in high income men.

The study contains less discussion of the impact on women, due to their lower hospitalization rates. In the Taiwan NHI system, the insurance premium level paid by an unemployed individual may be determined by the incomes of his/her relatives who are employed. Due to the lower labor participation rate among women than among men, women receive less real income and were therefore excluded in our study. Nonetheless, similar trends (not significant) for women were observed in our extended analysis (data not shown).

## 5. Strengths and Limitations

Our study used an entire and meaningful subset of the population of Taiwan. To our knowledge, there have been few studies assessing the nationwide effects of economic crises in terms of alcohol-related morbidity. A few limitations of our study are present. First, in Taiwan, the domestic expenditure on cigarette and alcohol increased 3.4% between the periods of July 2007–June 2008 and July 2008–June 2009 [55]. However, complete and accurate monthly measures of the intervening factor, i.e., drinking consumption and behavior, are not available in our study, and a lack of measures for intervening factors lessens the plausibility of our findings. Second, while our time series models can control some of the confounding factors, the databases used in this study do not include more intimate data, such as actual income and wealth, and thus may not be able to better define the socio-economic condition of the individual. Related to this, the presumption in our study is that some men’s income levels fell with the recession. It should also be noted that large numbers of people can suffer individual economic shocks such as a job loss or eviction even in an economy not experiencing a recession. Conversely, even in an economy undergoing a recession, some people may not experience any drop in living standards [56]. With these points in mind, future studies focusing on the effects of economic factors at the individual level are suggested.

## 6. Conclusions

This study provides evidence that the economic recession and the reduction in the alcohol tax resulted in different effects on the levels of AADs in Taiwan among working-age men of different socioeconomic backgrounds. Low income men suffer from the highest probability of alcohol disorders when a recession hits or alcohol prices fall. Additionally, they take the brunt of the impact while the recession is reflected in the economic statistics, possibly because their employment situation is particularly vulnerable. The policy implication therefore, is that the government should prepare a better-knit safety net for times of recession. In Taiwan, remedial measures seemed to have responded to the need of the unemployed, which may have ameliorated the impact of the economic recession to a certain extent. Considering the differential impact on people of different income levels, these ameliorative measures may need to be designed to meet the needs of people of different socio-economic conditions.

## Figures and Tables

**Table 1:** Monthly alcohol-attributed incident rates of hospitalization (per 100,000 persons) working-aged men.

Year	Low Income		Middle Income		High Income	
	Rate	Change (%)	Rate	Change (%)	Rate	Change (%)
2007	78.3	-	60.4	-	18.6	-
2008	82.0	4.5%	57.8	−4.5%	18.1	−3.2%
2009	84.6	3.1%	54.3	−6.5%	19.9	9.1%
2010	92.6	8.6%	56.7	4.3%	23.4	15.3%
2011	98.1	5.7%	56.9	0.3%	26.8	12.4%
2012	105.1	6.7%	55.7	−2.1%	26.6	−0.7%

**Table 2:** Effects of the economic recession on monthly alcohol-attributed incident rates of hospitalization (per 100,000 persons) in working-aged men.

Income Groups	Economic Intervention in 2008					Tax Intervention in 2009					Box-Ljung
	ω ^a^ (SE)	T	*p*-Value	Mean Before First Intervention (1/10^5^)	Change ^b^ (%)	ω ^a^ (SE)	T	*p*-Value	Mean Before Second Intervention (1/10^5^)	Change ^b^ (%)	Q-Statistics (df ^c^)
	δ (SE)					δ (SE)					*p*-Value
Low	5.702 (1.349)	3.760	0.00017	80.189	7.11	0.997 (0.229)	4.354	<0.0001	83.628	39.74	22.82 (17)
	-	-	-	-	-	970 (0.017)	57.06	<0.0001	-	-	0.155
Middle	1.042 (0.329)	3.167	0.0015	59.870	−22.90	-	-	-	54.745	-	15.54 (16)
	0.924 (0.015)	18.12	<0.0001	-	-	-	-	-		-	0.485
High	-		-	17.882	-	0.561 (0.267)	2.101	0.0356	19.283	47.69	12.01 (16)
	-	-	-	-	-	0.939 (0.047)	19.98	<0.0001	-	-	0.743

^a^ Difference of adjusted incidence rate of hospitalization for alcohol-attributed diseases (per 100,000 persons) was estimated with a monthly autoregressive integrated moving average model. ^b^ Percent change $\frac{\omega }{1-\delta }$ in the outcome based on the average of the 12 months immediately prior to the two interventions. ^c^ Degree of freedom. -:No significant effect of the interventions was observed (data not shown).
